# Terminal differentiation and anti-tumorigenic effects of prolactin in breast cancer

**DOI:** 10.3389/fendo.2022.993570

**Published:** 2022-09-08

**Authors:** Suhad Ali, Dana Hamam, Xueqing Liu, Jean-Jacques Lebrun

**Affiliations:** Department of Medicine, Cancer Research Program, The Research Institute of the McGill University Health Centre, McGill University, Quebec, Canada

**Keywords:** Prolactin/prolactin receptor, breast cancer, stem cells, plasticity, single cell analysis, JAK/STAT, differentiation

## Abstract

Breast cancer is a major disease affecting women worldwide. A woman has 1 in 8 lifetime risk of developing breast cancer, and morbidity and mortality due to this disease are expected to continue to rise globally. Breast cancer remains a challenging disease due to its heterogeneity, propensity for recurrence and metastasis to distant vital organs including bones, lungs, liver and brain ultimately leading to patient death. Despite the development of various therapeutic strategies to treat breast cancer, still there are no effective treatments once metastasis has occurred. Loss of differentiation and increased cellular plasticity and stemness are being recognized molecularly and clinically as major derivers of heterogeneity, tumor evolution, relapse, metastasis, and therapeutic failure. In solid tumors, breast cancer is one of the leading cancer types in which tumor differentiation state has long been known to influence cancer behavior. Reprograming and/or restoring differentiation of cancer cells has been proposed to provide a viable approach to reverse the cancer through differentiation and terminal maturation. The hormone prolactin (PRL) is known to play a critical role in mammary gland lobuloalveolar development/remodeling and the terminal differentiation of the mammary epithelial cells promoting milk proteins gene expression and lactation. Here, we will highlight recent discoveries supporting an anti-tumorigenic role for PRL in breast cancer as a “pro/forward-differentiation” pathway restricting plasticity, stemness and tumorigenesis.

## Introduction

Cancer is a complex disease caused by both genetic and epigenetic mutations/alterations promoting uncontrolled growth and ultimately ensuring the dysregulation of control mechanisms of normal tissue differentiation and homeostasis (1, 2). Recent advances in our understanding of the process of tumorigenesis have indeed emphasized tumor plasticity (encompassing dedifferentiation, blocked differentiation, and/or trans-differentiation) and enrichment of stem-like cell population(s) underlie tumor heterogeneity, progression and therapy failure and resistance. Just recently, tumor cellular plasticity was recognized within the “hallmarks” of cancer, initially proposed in 2000, as an enabling feature promoting tumor evolution and progression (2, 3). Thus, reprograming and/or restoring differentiation of cancer cells has been proposed to provide a viable approach to reverse the cancer phenotype through differentiation and terminal maturation (4). Importantly, while differentiation-based therapeutic approaches have already been employed and shown success in the treatment of hematological malignancies, their application to solid tumors including breast cancer is yet to be fully developed and is an area of intense investigation (5–8). Thus, it is evident that characterizing mechanisms/pathways promoting differentiation in breast cancer is fundamental and will help generate novel differentiation-based reagents and approaches to better manage and serve patients stricken by this aggressive disease. In this review we will summarize knowledge gained from exploring the impact of the mammary differentiation hormone PRL in the context of suppression of breast tumorigenesis through restoration of differentiation and suppression of stemness.

## Breast cancer differentiation state illustrates good prognosis vs poor prognosis

Tumor differentiation state in breast cancer is classically determined by the tumor grade established based on the use of certain histological and morphological criteria, such as nuclear pleomorphism, gland or tubule formation and number of dividing cells, and has long been used as predictive of cancer behavior where immature tumor (not resembling the tissue of origin) is more aggressive than the more differentiated counterpart (9–11). Findings emanating from a large study examining tumor grade and patient outcome indicated that high-grade (grade 3) breast cancers tend to recur and metastasize early following diagnosis and show poor prognosis, whereas low-grade tumors (grade 1) tend to show a very good outcome and grade 2 tumors show an impaired outcome in the long term (12, 13).

Moreover, the correlation of breast cancer differentiation state with tumor behavior and patient outcome can also be gleaned from the current classification schemes of breast cancer whether based on evaluating the histological expression of the estrogen receptor (ER), progesterone receptor (PR) and human epidermal growth factor receptor-2 (HER2), or classifications based on intrinsic gene expression and genomic profiling (PAM 50) (14, 15). Largely, breast cancers can be categorized into molecularly distinct subtypes including, luminal A, luminal B, HER2-enriched (HER2-E) and basal-like and claudin low (representing triple negative breast cancer [TNBC]: ER^-^, PR^-^, HER2^-^) (16–18). Among the different breast cancer subtypes, the most differentiated breast tumors are those of the luminal A subtype which tend to be of low grade showing epithelial-like differentiation and interestingly have the least aggressive tumor biology and the most favorable prognosis. In contrast, luminal B, HER2-E and TN are considered ‘aggressive’ subtypes, characterized by a tumor biology showing generally high grade, high mitotic/proliferation index, and a greater risk of local recurrence, metastasis and poor survival outcomes (19–21). In agreement, recent studies using single cell (sc) approaches have further emphasized the phenotypic and cellular diversity of breast tumors (22, 23). Importantly, tumor cellular phenotypic abnormalities linked to deviation from the juxta-tumoral area were found to be higher for tumor cells of luminal B, luminal B-HER2+, TN, and grade 3 tumors than for luminal A and lower grades tumors. Moreover, phenotypically abnormal cells were also correlated with hypoxic phenotype and proliferation marker expression which were previously linked to poor differentiation in breast cancer (24). Moreover, sc-analyses of the heterogeneous TNBC subtype showed that TNBC tumors of the basal-like phenotype as exhibiting high proliferation index compared to the TNBC subtype showing luminal-androgen receptor (LAR)-differentiation phenotype (23, 25).

Additionally, over the past two decades studies evaluating the breast cancer cell-of-origin and the cancer stem cell hypothesis have emphasized a link between the mammary stem cell (MaSC) hierarchy, breast cancer stem cells (BCSCs) and the inter- and intra-tumoral heterogeneity of breast cancer (26–28). These studies highlighted that essentially breast cancer originate from a mammary luminal progenitor population and indicated the presence of rare populations of cancer cells within breast tumors that exhibit high tumorigenic capacity and resistance to chemotherapy with a stem-like phenotype capable of self-renewal and tumor repopulation. These aggressive BCSCs are found to be mostly enriched in the aggressive breast tumors such as TNBC as well as HER2-E tumors (29). In summation, there is extensive literature implicating loss of tumor differentiation, and the accumulation of dedifferentiated immature cancer cells endow breast cancer with aggressive features and is predictor of poor prognosis.

## PRL regulation of alveolar differentiation and apical/basal polarity

**Figure 1:**
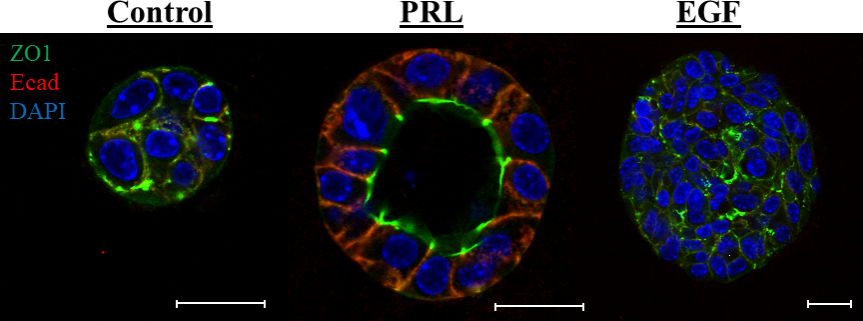
PRL induces mammary A/B polarity and acini morphogenesis: Primary mouse mammary epithelial cells grown in 3D culture were stained with antibody to ZO1 (green) and Ecad (red). Nucleus was counter stained with DAPI (blue). Scale bar, 20 µm. The morphology of the colonies was evaluated following different treatments: (1) Control: 2% FBS, (2) PRL: 2% FBS + 2 µg/mL ovine PRL or (3) EGF: 2% FBS + 10 ng/mL EGF. In contrast to control or EGF treated cells, PRL treated mammary epithelial cells organize around a single lumen showing apical localization of the tight junction protein ZO1 and basal/lateral localization of the adhesion protein E-cadherin.

The hormone PRL is best known as a lactation hormone critical for mammary gland lobuloalveolar development/remodeling and the terminal differentiation of the mammary epithelial cells promoting milk proteins gene expression and lactation (30–32). PRL mediates its effects by binding to its specific receptor (PRLR), resulting in receptor dimerization and activation of different intracellular signaling cascades, most well studied is the Jak2/Stat5 pathway (33). Importantly, PRL, PRLR, Jak2 and Stat5 knockout mouse models have all shown defects in mammary gland development and lactation, clearly highlighting the prominent role of PRL in the normal development and functional differentiation of the mammary gland (34–38). Indeed, during the pregnancy/lactation cycle the mammary gland undergoes a complex growth and remodeling characterized by the establishment of the secretory alveolar units. These mammary alveoli consist of a layer of terminally differentiated luminal mammary epithelial cells attaining apical/basal (A/B) polarized architecture with closed tight junctions and well-established adherence junctions. Their main function is to allow for the synthesis and directional secretion of milk proteins and solutes into the lumen of the alveolar unit to the mammary ductal system upon suckling of the infant (39, 40). In agreement with the above work and crucial to the differentiation role of PRL in the breast, using a well-established *ex vivo* mammary 3D cell culture model, PRL signaling through Jak2 was found to induce A/B polarity and to organize the mammary epithelial cells around a single hollow lumen (41, 42). Recently, PRL regulated gene Pre-B-Cell Leukemia Transcription Factor-Interacting Protein 1 (PBXIP1/HPIP) was also found to play a role in PRL-mediated mammary epithelial cell differentiation and acini morphogenesis (43). Moreover, studies from our laboratory also highlighted that PRL indeed limits the proliferative capacity of the mammary epithelial cells and provided resistance to the proliferative effects of EGF (42, 44). Previously, PRL was shown to be part of a cooperative signaling network with EGF promoting alveolar survival, morphogenesis, and functional differentiation (45, 46). Our studies however, highlighted an important negative cross-talk between PRL/Jak2-differentiation axis and the EGF-Erk1/2-proliferative pathway (44). Together, these results expand on the vital role for PRL in deriving the normal differentiation program of the mammary cells and constrains the proliferative effects of growth factors (**Figure 1**).

## PRL regulation of the MaSC hierarchy and terminal differentiation

Extensive research has been devoted to characterizing the breast epithelium delineating the mammary stem cell (MaSC) hierarchy and its relevance to breast cancer inter-tumor heterogeneity with the interest of identifying new therapeutic targets in breast cancer (27, 47). Studies have described a MaSC hierarchy consisting of different cell populations based on expression of cell surface markers into: basal (EpCAM^low/-^/CD49f^high/+^), luminal progenitor (EpCAM^high/+^/CD49f^high/+^), and mature luminal cells (EpCAM^high/+^/CD49f^low/-^) (48). With advances in sc-analyses, recent studies have indeed expanded on this model and highlighted a more complex mammary lineage hierarchies and cell states within the mammary epithelium (49–51). Still, these studies confirmed that the epithelium in mouse and human samples are mainly divided into three major clusters, namely basal cells, luminal progenitors, and mature hormone-sensing luminal cells. We previously investigated the contribution of PRL to the differentiation program of the MaSC hierarchy. Mammary epithelial cells isolated from mid-pregnant mice showed two distinct cellular sub-populations based on the expression profile of EpCAM and CD49f. One population featured a surface marker signature with EpCAM^high/+^/CD49f^high/+^ defining the luminal progenitor cells and another with EpCAM^high/+^/CD49f^low/-^ defining mature luminal cells. Comparing with EGF treated cells, treatment with PRL resulted in a shift in the luminal progenitor (EpCAM^high/+^/CD49f^high/+^) cells into the mature luminal (EpCAM^high/+^/CD49f^low/-^) cells suggesting that PRL derives the terminal differentiation of the mammary epithelial cells (42). This proposition is also supported by the sc-studies described above where PRLR expression was found to be enriched in the most differentiated hormone sensing cells and least expression was found in the basal compartment (49). As well, PRL-target milk proteins (e.g. Wap, Csn2) were expressed exclusively in cellular clusters composed of cells from gestation and lactation defining them as differentiated secretory alveolar cells. Interestingly, Assay for Transposase-Accessible Chromatin (ATAC) analyses pointed to a strong correspondence between high FOXA1 transcription factor, known regulator of luminal differentiation and an antagonist of the epithelial-to-mesenchymal transition (EMT), motif accessibility, and gene expression in the hormone-responsive luminal cells (52). Interestingly, we have previously found that there is positive correlation of expression between PRLR and FOXA1 in breast cancer cases (53). Altogether, these results suggest that PRL/PRLR derives the terminal maturation of the mammary stem cells into a differentiated hormone sensing cells and differentiated alveolar cells. These results also highlight the close association between FOXA1 and the PRLR in the differentiated hormone sensing luminal cells that is maintained in breast cancer.

## Evidence of anti-tumorigenic functions of PRL/PRLR pathway in breast cancer

While the role of PRL as a differentiation factor in the mammary gland is well known, its role in breast cancer is still not fully characterized. Several studies using *in vitro* cell culture approaches as well as transgenic and knock-out mouse models have highlighted a pro-tumorigenic role for PRL in breast cancer promoting tumor initiation, development and metastasis (reviewed elegantly in this series Schuler, LA and O’Leary, KA as well as previously (54)). These findings prompted interest in developing strategies to block PRL as a treatment modality in breast cancer. Most recent and indeed direct approach was the generation of humanized antibodies to block PRLR as a targeted therapy in breast cancer (55, 56). Following extensive characterization of these antibodies, their therapeutic value was assessed. Indeed, these agents failed to show any antitumorigenic effects in a landmark multicenter clinical trial performed in PRLR expressors breast cancer patients (Novartis, 2016) (USA, Belgium, Italy and Spain), despite effective blockage of the PRLR, resulting in the termination of the trial (57, 58). The lack of anti-tumorigenic effects of blockers of PRLR suggests that the described pro-tumorigenic role of PRL in breast cancer BC is not of clinical value. Also, these results indicate that PRL role in breast cancer needs further evaluation.

Epidemiological studies examining the normal physiological levels of circulating PRL (2-29 ng/mL) have implicated PRL as a risk factor and is involved in breast cancer etiology (59–63). However, later extended follow-up analyses showed either modest association, that is limited to patients who were on hormone replacement therapy or no significant associations (60, 61, 64, 65). Importantly, no differences in mean serum PRL levels in premenopausal (~21 ng/mL) or postmenopausal (~13 ng/mL) breast cancer cases compared with normal cases was reported (65). This finding suggests that serum PRL is not a breast cancer risk factor. In addition, studies of patients with conditions that result in high circulating PRL levels such as prolactinomas or the use of antipsychotics showed no causal link to breast cancer (66, 67). In fact, other conditions that lead to high circulating levels of PRL (~200 ng/mL) such as breastfeeding have been linked to reduced risk of breast cancer. A seminal study (2002) that examined 50,000 breast cancer cases from 47 epidemiologic studies in 30 countries, reported that the relative risk for breast cancer is reduced by 4.3% for every 12 months a woman breastfeed (68). Another study reported a 14-28% lower risk of developing breast cancer in parous women who ever breastfed compared with parous women who never breastfed (69). Furthermore, little-to-no breastfeeding correlated with increased risk of developing aggressive types of breast cancer (70–72). While studies have emphasized the local/autocrine PRL and not the circulating endocrine PRL as contributing to mammary tumorigenesis and breast cancer development, however, other studies using large breast cancer patient data and cell lines provided different conclusions. PRL mRNA expression was found to be either very low or undetectable in the majority of samples representing 144 breast cancer patients and in many breast cancer cell lines and the study concluded that autocrine PRL signaling is unlikely to be a general mechanism promoting tumor growth in breast cancer (73). We have also analyzed PRL protein and mRNA levels in breast cancer cases (74). Interestingly, our results agreed with the above report and showed a significant down regulation of PRL expression in breast cancer compared to normal tissue. Moreover, inline with the differentiation role of PRL in the breast, expression of PRL mRNA was associated with more differentiated tumors, early stage, smaller tumor size and absence of distant metastasis with higher PRL mRNA levels correlating with prolonged relapse free survival (74). Importantly, in preclinical xenograft mouse models of TNBC and HER2-E breast cancer types, PRL was found to cause tumor downstaging as measured by tumor volume/growth and expression of the proliferative marker Ki67 (53, 75, 76). Also, PRL was found to suppress induction of the cytokeratin-5 (CK5)-positive stem-like population in breast cancer cells both *in vitro* and *in vivo* (77, 78). As well, PRL was recently found to sensitize ER+ breast cancer cells to tamoxifen in a xenograft mouse model expressing hPRL gene (79). Altogether, these findings implicate that PRL of endocrine or tumor source is not a risk factor in breast cancer but rather a marker of more differentiated and less aggressive tumors and is a potential therapeutic agent.

Assessing the expression levels of PRLR in breast cancer cases is vital to further define the role of PRL in breast cancer. Whereas short forms of the human PRLR generated by alternative splicing or as mutant truncation forms have been described, the long form of the PRLR is considered as the signaling hub for PRL (80–82). Previous reports have examined PRLR expression and have reported a widespread expression in breast cancer samples (83). More recent findings contradict these observations and implicate that PRLR expression is generally downregulated in breast cancer. For example, it was reported that using specific anti-human PRLR antibodies in a screen of 160 mammary adenocarcinomas demonstrated significant immunoreactivity in only 4 tumors (ie less than 3% expression). This led the authors to conclude that PRLR is generally not strongly upregulated in human breast cancer (84). We previously used human breast cancer cases organized in tissue microarrays as well as bioinformatics analyses and datasets to assess the expression of PRLR in breast cancer. We found that PRLR expression to be significantly downregulated in invasive breast cancer, only 21% of invasive cases showed detectable expression of the PRLR in comparison with normal/benign (80%) and *in situ* carcinoma (60%) (85). In addition, gene expression level of PRLR was also evaluated in relation to intrinsic molecular subtypes, tumor grade, and patient outcome using GOBO database for 1881 breast cancer patients. PRLR expression was found to associate with less aggressive clinicopathological parameters such as lymph node negativity and low-grade well-differentiated tumors. Also, among the different breast cancer subtypes, PRLR mRNA levels were highest in luminal A subtype and least expression was detected in the most aggressive TNBC basal-like subtype. Furthermore, PRLR expression was significantly associated with better survival outcome in breast cancer cases (85). Interestingly, within the TNBC subtypes, PRLR gene expression positively correlated with luminal and epithelial metagenes (LAR and Epithelial Cell-Cell adhesion), whereas it negatively correlated with metagenes defining the aggressive TNBC basal-like (BL) and mesenchymal stem-like subtypes (MSL) (25, 53). A subsequent study also found that PRLR expression defined a patient population with better prognosis showing lower recurrence and higher overall survival in TNBC patients (86). Interestingly, reports have shown that expression of truncated forms of the PRLR long form resulted in initiation of mammary tumorigenesis in mouse models of ER+ breast cancer as well as in human MCF10A xenograft model (87, 88). Similarly, direct knock out of the PRLR in ER+ and HER2-E breast cancer cell lines led to enhanced tumorigenic and metastatic phenotype as well as resistance to conventional therapies (89). Altogether, these results implicate that loss of PRL/PRLR expression contributes to the initiation and progression of breast cancer and argues against a role for PRL/PRLR in promoting breast tumorigenesis.

In agreement with the above data showing PRL/PRLR as favorable markers of tumor differentiation and suppressors of tumorigenesis, other groups have demonstrated that expression/activation of the PRL effector molecule-Stat5a in breast cancer promotes adhesion and inhibits invasion of breast cancer cells (90). As well, Stat5a expression in breast cancer clinical cases was found to associate with histologic differentiation (low grade) and favorable prognosis, whereas loss of Stat5a expression was associated with tumor progression, unfavorable prognosis and increased risk of failure to antiestrogen therapy (90–94). Recently, Stat5a-N-myc interactor (NMI)-signaling also further supported an anti-tumorigenic role for Stat5a. It was reported that this signaling axis is downregulated in breast cancer and its expression is distinctive for less frequent metastasis and good prognosis (95). Additionally, examining expression of PRL signaling pathway-based gene signature composed of PRL, PRLR, Jak2 and Stat5a showed a significant association with more differentiated tumors and prolonged survival (74). Interestingly, PRL-responsive milk proteins were also shown to inhibit tumorigenesis and invasion of breast cancer cells (96–98). Moreover, global gene profiling of prolactin-modulated transcripts in ER+ human breast cancer xenotransplant model revealed that PRL-upregulated genes were enriched in pathways involved in differentiation and a gene signature based on PRL-upregulated genes was associated with prolonged relapse-free and metastasis-free survival in breast cancer patients (99). Interestingly, gene profiling of PRL stimulated mammary epithelial cells also defined a gene signature derived from PRL-upregulated target genes to be associated with well differentiated tumors, whereas expression of a gene signature composed of PRL-downregulated genes showed a significant association with shortened distant metastasis free survival (74). Importantly, functional investigations of these PRL-downregulated genes identified novel players in breast cancer. Indeed, PRL-downregulated genes were found to be drivers of oncogenic processes including the epigenetic A-to-I RNA editing process and the metastatic and stemness epithelial-mesenchymal-plasticity (EMP) process (77, 78, 100, 101). Altogether, there is now a large body of evidence implicating PRL/PRLR pathway as a clinically relevant anti-tumorigenic pathway in breast cancer.

## PRL/PRLR and the cancer cell-of-origin

**Figure 2:**
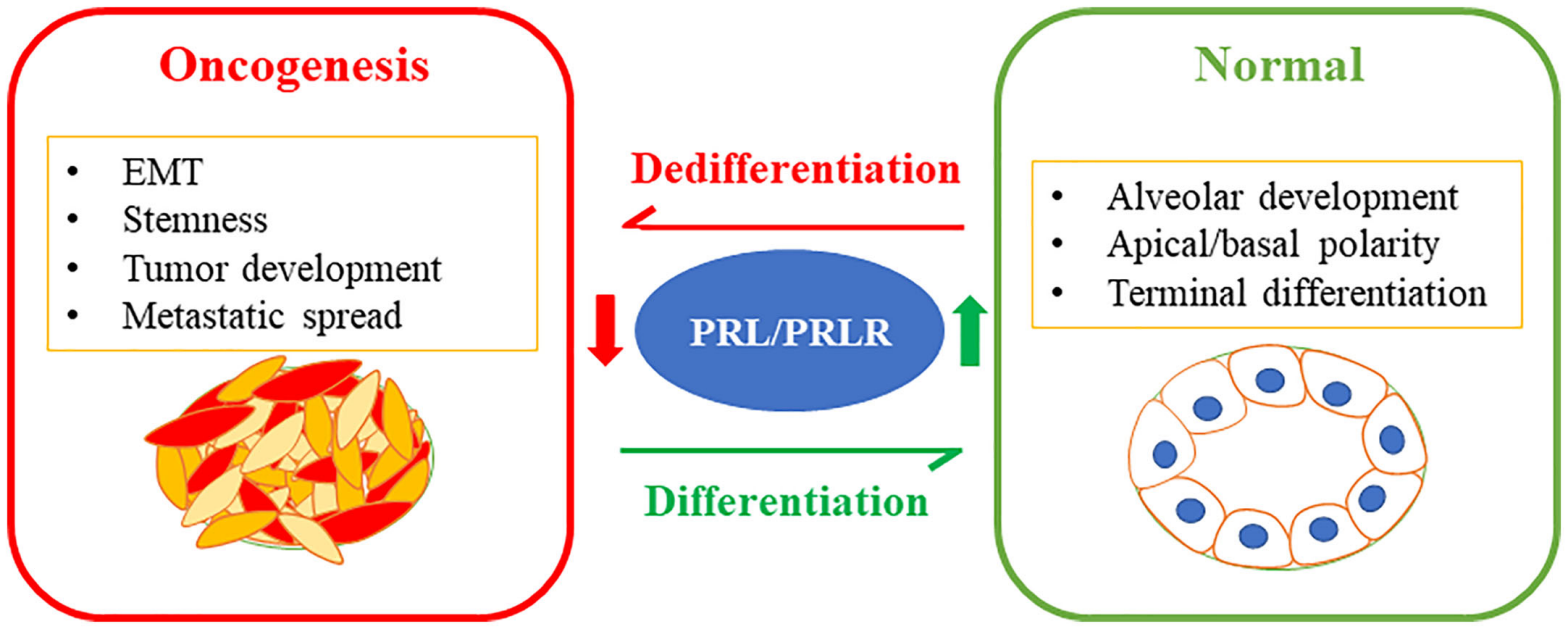
PRL/PRLR signaling pathway in breast cancer differentiation limiting tumorigenesis: The PRL/PRLR pathway is a fundamental pathway promoting mammary gland development, morphogenesis, and terminal differentiation of the mammary epithelial cells. Loss of this hormonal pathway is a marker of aggressive breast cancer characterized by poor differentiation promoting stem-like phenotype, tumor development and metastatic spread.

The molecular classification of breast cancer subtypes based on global gene expression profile had a fundamental impact on the current understanding of inter-tumor heterogeneity. Studies have also highlighted the link between the mammary stem cells hierarchy serving as the cell of origin for malignant transformation giving rise to the various tumor subtypes (16, 17, 102, 103). Direct comparison of the gene expression profiles of normal mammary epithelial subsets described above (i.e. basal/MaSC, luminal progenitor, and mature luminal cells) to those of breast tumors based on the molecular subtype classifications were performed (104). Interestingly, luminal A and B subtypes showed high similarity to the mature luminal cell population EpCAM ^high/+^/CD49f^low/−^. The luminal progenitor gene expression signature was very similar to the basal-like subtype showing expression of basal-like markers; including cytokeratins14 and 5/6 (105). On the other hand, the MaSC-signature exhibited high association with the claudin-low subtype (106). Clinically, the detection of EpCAM^low/-^/CD49f^high/+^ in breast tumors was shown to be associated with poor clinical prognosis (107). Studies have also linked the MaSC hierarchy with the profile of tumor initiating cells/BCSCs characterized by CD44^+^/CD24^-^ and ALDH^+^, where, CD44^+^/CD24^-^ correspond to the MaSC population (EpCAM^low/-^/CD49f^high/+^) and ALDH^+^ correspond to the luminal progenitor (EpCAM^high/+^/CD49f^high/+^) cells (29). Moreover, activation of the EMT program is well known to be a deriver of phenotypic plasticity and stemness in breast cancer (108, 109). Interestingly, our original work investigating the role of PRL in breast cancer BC revealed PRL to act a potent suppressor of the EMT process, further inhibiting the invasive capacity of breast cancerBC cells. This effect of PRL was found to be linked to the negative-crosstalk between PRL-induced signaling cascade and the two major pro-metastatic pathways MAPK-Erk1/2 and TGFβ (110). Subsequently, we have accumulated compelling evidence and notably, we found that treatment of breast cancer cells representative of the TNBC subtype or of the HER2-E subtype significantly depleted the highly tumorigenic CD44^+^/CD24^−^ and ALDH^+^ BCSC subpopulations and induced their differentiation into the least tumorigenic phenotype (ie CD44^−^/CD24^−^ and ALDH^-^ resulting in suppression of their tumorsphere formation/self-renewal capacities (53, 76). On the other hand, loss of expression of the PRLR in ER+ and HER2-E breast cancer cells resulted in the enrichment of these BCSC populations. Clinically, *Prlr* gene expression was also found to have inverse relationship with *CD44* gene expression in TNBC patients (76). Moreover, in RNA-seq data of breast cancer patients, PRLR expression correlated negatively with the mRNA levels of a number of genes (including *Aurkb*, *Ccna2*, *Scrn1*, *Npy*, *Atp7b* and *Chaf1b*) that are related to stemness, resistance to therapy and poor patient outcome (111). Among the multiple isoforms of ALDH, ALDH1A1 and ALDH1A3 are known to be associated with cancer stem cells (112, 113). Interestingly, PRL treatment of HER2-E breast cancer cells was found to suppress the expression levels of both ALDH1A1 and ALDH1A3 mRNA expression. Recent sc- analyses of mammary epithelial cells also identified ALDH1A3 as a marker of luminal progenitor cells having its levels gradually decreased as cells progressed away from their common origin and differentiated to express higher levels of PRLR either in the in hormone sensitive differentiated cells or the alveolar differentiated trajectories (49). In summary, PRL imparts significant anti-tumorigenic effect in breast cancer through differentiation and terminal maturation (**Figure 2**).

## Outlook

In view of our improved understanding of the contribution of tumor cellular plasticity and loss/defects in normal tissue differentiation mechanisms to cancer progression and tumor evolution, significant efforts are directed at exploiting differentiation pathways as therapeutic avenues in cancer. The premise of differentiation therapy (DT) in cancer is a strategy that aims at engaging-forward differentiation and cellular reprograming restricting the proliferative, tumor repopulation, stemness, EMT and metastatic capacities of tumor cells leading to the cessation of the aggressive tumor phenotype and offering the cancer patients improved survival for decades (6, 114–116). Interestingly, the concept of DT was first proposed by Pierce in 1961, reporting on the differentiation of aggressive forms of teratocarcinoma into benign forms and in 1984 was the first clinical application of DT when the use of all-trans retinoic acid was approved for acute promyelocytic leukemia (117). Currently, still under development, several highly promising candidate differentiation and cellular reprograming targets encompassing epigenetics, transcription factors, metabolic and modulators of the cancer stem cells are being evaluated preclinically and clinically as anti-cancer therapeutics (i.e., inhibitors of histone deacetylases (HDACi) (118), micro-RNAs (119) peroxisome proliferator-activated receptor-γ (PPARγ) pathway (120–122), inhibitors of bromodomain-containing protein 4 (BRD4i) (123) among others (115)). Indeed, whereas significant advances have been achieved in treatment options for patients with hormone receptor positive tumors including anti-endocrine-based therapies, and more recently CDK4/6 inhibitors (124), and for HER2-E subtype targeting HER2 (trastuzumab (Herceptin), lapatinib, pertuzumab and trastuzumab emtansine TDM-1) no effective treatment options besides chemotherapy is available for patients with TNBC (125, 126). Notably, none of these approaches are differentiation-based therapeutics. Therefore, identifying drivers and mechanisms of tumor cellular differentiation in breast cancer are urgently in need in our pursuit to limit aggressive malignant changes of tumor progression and to develop new generation of biomarkers and anti-cancer therapies centered on the “pro/forward-differentiation” concept. Collectively, in breast cancer accumulating data implies PRL/PRLR as a clinically relevant potent differentiation pathway limiting the tumorigenic phenotype and thus may serve as a potential pro-differentiation therapeutic candidate.

## Author Contributions

SA: formulation and writing of the manuscript DH: contributed to the writing of the manuscript and generated **Figure 2** XL: Contributed to the writing of the manuscript and generated **Figure 1** JJL: Contributed to the formulation and editing of the manuscript. All authors contributed to the article and approved the submitted version.

## Funding

This work is supported by the Canadian Institutes of Health Research (CIHR-Funding Reference Number-PJT-173413). Dana Hamam is a recipient of the Fonds de Recherche du Québec (FRQS) Studentship award.

## Conflict of interest

The authors declare that the research was conducted in the absence of any commercial or financial relationships that could be construed as a potential conflict of interest.

## Publisher’s note

All claims expressed in this article are solely those of the authors and do not necessarily represent those of their affiliated organizations, or those of the publisher, the editors and the reviewers. Any product that may be evaluated in this article, or claim that may be made by its manufacturer, is not guaranteed or endorsed by the publisher.

## Abbreviations

A/B, apical/basal; ALDH; aldehyde dehydrogenase gene; ATAC, Assay for Transposase-Accessible Chromatin; BCSCs, breast cancer stem cells; BL, basal-like; BRD4i, bromodomain-containing protein 4 inhibitor; CK5, cytokeratin-5; DT, differentiation therapy; EGF, epidermal growth factor; EMP, epithelial-mesenchymal-plasticity; EMT, epithelial-to-mesenchymal transition; ER, estrogen receptor; GOBO, Gene expression- based Outcome for Breast Cancer Online; HDACi, histone deacetylases inhibitor; HER2, human epidermal growth factor receptor-2; HER2-E, HER2-enriched; hPRL, human prolactin; LAR, luminal-androgen receptor; MaSC, mammary stem cell; NMI, N-myc interactor; PPARγ, peroxisome proliferator- activated receptor gamma; PR, Progesterone receptor; PRL, Prolactin; sc, single cell; TNBC, Triple negative breast cancer.
